# Magnitudes of diseases in dogs vary among different levels of age, gender, breed, and season: A hospital-based, retrospective cross-sectional study

**DOI:** 10.1016/j.heliyon.2021.e08287

**Published:** 2021-10-29

**Authors:** Mohammod Misbah Uddin, Himel Talukder, Obaidul Islam, Md. Asaduzzaman, Moumita Das, Md. Irtija Ahsan, Saiful Islam

**Affiliations:** a Department of Epidemiology and Public Health, Sylhet Agricultural University, Sylhet, 3100, Bangladesh; b Department of Livestock Production and Management, Sylhet Agricultural University, Sylhet, 3100, Bangladesh; c Central Veterinary Hospital, Dhaka, 1000, Bangladesh; d Department of Physiology, Sylhet Agricultural University, Sylhet, 3100, Bangladesh

**Keywords:** Magnitudes, Determinants, Dog diseases, Bangladesh

## Abstract

Dogs, the most common companion animal of humans, perform not only the auxiliary of an individual, but also contribute to the nations’ crime and defence departments. Knowing the determinant-based disease status of dogs is imperative to keep them healthy by subsequent prevention and control of those diseases; however, such baseline epidemiological information is limited. Therefore, a retrospective cross-sectional study was conducted to estimate the proportional incidence of dog diseases to explore their magnitudes, and we compared them to different levels of intrinsic (age, gender, breed) and extrinsic determinants (season). Purposively, data of a total of 1,557 cases of different diseases were collected from the record book of the Central Veterinary Hospital (CVH), Dhaka, Bangladesh. The proportional incidence was calculated as the proportion of cases of a specific disease among total number of cases of all types of diseases attended the hospital during the study period. Diseases of dog were categorized into infectious, non-infectious, and non-specific. Results showed that the highest proportional incidence was noted in infectious diseases (53.8%) followed by non-infectious diseases (23.4%) and the lowest in non-specific (22.7%) cases. Among them, disease-specific proportional incidence was remarkable in case of mange (9.5%), parvovirosis (8.7%), lacerated wound (8.5%), ectoparasitism (8.3%), helminthiasis (7.8%), and fracture (5.7%). The occurrence of mange varied significantly (p < 0.05) among all studied determinants (age, gender, breed, and season); while significant discrepancies (p < 0.05) in magnitudes of lacerated wound, dystocia, abortion, and gastroenteritis were observed among various groups of age, gender, and breed. Accordingly, dermatitis and orchitis had significant differences (p < 0.05) in proportional incidence amid various levels of age, gender, and season; whilst the burden of parvovirosis and alopecia differed significantly (p < 0.05) amongst different categories of age, breed, and season. The magnitude of otorrhoea showed a significant (p < 0.05) variation among different groups of gender, breed, and season. The proportional incidence of other diseases also varied significantly (p < 0.05) amongst either one or two studied determinants. This study provides a valuable insight about important diseases in dogs, which may serve as useful baseline information for disease prioritization and subsequent planning of effective control and prevention measures against those diseases.

## 1 Introduction

Dogs, upon appearing on the earth about 20 million years ago, have become the most successful canid and have attained the adaptation of human habitation worldwide (Sharma et al., 2008). Individuals keep dogs for companionship and recreational purposes where they play valuable role in the physical, mental, and social wellbeing of their owners, particularly for the children (Dohoo et al., 1998). Their amenable attitude, ecstatic behavior, submissive manner, and natural instinct to their owners have underscored their acceptance as a pet animal popularly (Robertson et al., 2000). Besides, dog is known to be a very intelligent animal that can detect human psychology easily, and even a well-trained dog can be used as a substitute of a qualified person to perform certain specific crucial functions (Duranton et al., 2015). In many countries, aside from recreational purposes or companionship, dogs are also used to serve for the nation's defence department where they play a pivotal role to detect crimes and criminals (Islam et al., 2019).

In Bangladesh, BGB (Border Guards Bangladesh), RAB (Rapid Action Battalion), and the Police department maintain dog squads to assist in the investigation of crimes in anti-narcotic, anti-bombing, and other related contexts, and the dog breeds they use for these purposes are mainly German Shepherd, Dutch Shepherd, and Retriever (Adrija, 2020). Keeping pet dogs is more popular in the Dhaka city than other cities of the country, and the owners do their utmost care for the betterment of their pets by proper feeding, care, management, and regular health check up by the veterinarians; notwithstanding they are not free from the diseases (Islam et al., 2019).

The common diseases of dogs can be categorized into infectious, non-infectious, and non-specific diseases (Hossain et al., 2017). Among infectious diseases, dogs are mostly prone to parvovirosis, distemper, salmonellosis, helminthiasis, mange, myiasis, and pneumonia; while fracture, lacerated wound, abortion, dystocia, and vitamin deficiency are the most common non-infectious cases; and the most frequent non-specific cases are gastroenteritis, otorrhoea, dermatitis, and alopecia (Shima et al., 2015a).

A good knowledge of the epidemiology of these diseases of dogs is important for their prevention and control; however, such evidence-based information is limited. An epidemiological study providing information on disease status and associated determinants could be helpful for strategic prevention and control planning. Therefore, a study was conducted to estimate the proportional incidence of dog diseases; thus investigating the determinant-based magnitudes of those diseases.

## 2 Materials and methods

### 2.1 Study design and data collection

A retrospective cross-sectional study was designed to estimate the proportional incidence of different dog diseases to know their magnitudes. The Central Veterinary Hospital (CVH) of the Dhaka city is the most reliable source of information about pet animal diseases, as it is the centre for providing the veterinary services to the city dwellers rearing domestic and pet animals. Prior to this study, authorizations for investigations were obtained from hospital authorities on the condition that data would be treated anonymously. Therefore, four years' (January 2015 to December 2018) of patients data (Supplementary File 1) consisting of a total of 1,557 dog cases along their demographic characteristics (age, gender, and breed) were collected from the hospital's record book, maintained by the veterinary surgeons. The present study neither had any direct involvement in handling the sick dogs nor collecting the samples from them for the diagnosis. The hospital authority diagnosed the diseases for treatments and we only collected those data. Hence, the ethical approval was not applicable for this study.

### 2.2 Diagnosis of the diseases

In the hospital, diseases were diagnosed based on history, clinical signs and symptoms, and physical examinations. Moreover, blood, faeces, skin scrapping, nasal secretion, ear swab, and cerebrospinal fluids were sent to the adjacent Central Disease Investigation Laboratory (CDIL) for the confirmatory diagnosis of different recorded diseases. Polymerase chain reaction (PCR), rapid kit diagnosis, culture and microscopic examination of faeces, blood, ear swab, and skin strapping were major laboratory examinations. Besides, auscultation, x-ray, ultrasounds, and ultrasonography were performed for the confirmatory diagnosis, if needed.

### 2.3 Data entry, management, proportional incidence calculation and data analysis

The data were entered into a Microsoft Excel spreadsheet, and subsequently were organized and processed for further analysis. The diseases of dog were categorized into infectious, where causal agents were viruses, bacteria, and parasites; non-infectious, caused by various types of injury, accidents, vitamin/mineral deficiency, and hormonal imbalance; and the non-specific diseases like gastroenteritis, dermatitis, arthritis, orchitis, otorrhoea, alopecia, and poisoning as described by Hossain et al. (2017). The season was categorized as winter (November–February), summer (March–June), and rainy season (July–October), as described by Chowdhury et al. (2020); whereas, dogs were considered as puppy (0–7 months), adolescent (8–18 months), and adult (>18 months) according to their age, as suggested by Shima et al. (2015b). The data were then expressed as proportional incidence, which was calculated according to the following formula, described by Uddin et al. (2010).

$$\text{Proportional ​incidence}=\frac{\text{Total}\;\text{number}\;\text{of}\;\text{cases}\;\text{of}\;\text{a}\;\text{specific}\;\text{disease}\;\text{in}\;\text{the}\;\text{study}\;\text{period}}{\text{Total}\;\text{number}\;\text{of}\;\text{cases}\;\text{of}\;\text{all}\;\text{types}\;\text{of}\;\text{diseases}\;\text{recorded}\;\text{in}\;\text{the}\;\text{study}\;\text{period}}\text{X}100$$

Here, the number of cases in the numerator and denominator is the number of dogs that had at least one veterinary care event for the respective diagnostic categories. The calculated proportional incidence was used to explore the magnitudes of diseases. The variations in magnitudes among different levels of determinants (age, gender, breed, and season) were determined using chi-squared statistics, considering the P-value less than 0.05 as a level of significance. The analyses were performed through SPSS version 20 (SPSS, Inc., Chicago, IL).

## 3 Results

The study was focused on the investigation of the proportional incidence of dog diseases to estimate their magnitudes, and exploration of their distribution according to age, gender, breed, and season. The study revealed the highest proportional incidence in case of infectious diseases (53.8%) followed by non-infectious (23.4%) and non-specific diseases (22.7%). Among infectious diseases in terms of attributing pathogens, the magnitude of parasitic diseases was the highest (28.2%) followed by bacterial diseases (13.9%) and viral diseases (11.6%). The current study revealed parvovirosis (8.7%) as the most prevalent viral disease; while pneumonia (5.5%), colibacillosis (4.4%), and salmonellosis (4.0%) were commonly occurring bacterial diseases. Besides, mange (9.5%) and ectoparasitism (8.3%) were found as two noticeable parasitic diseases. Moreover, among the non-infectious diseases, the proportional incidence of lacerated wound (8.5%), fracture (5.7%), and vitamin deficiency (5%) were high, whilst gastroenteritis (5.5%), dermatitis (4.4%), and alopecia (4.1%) were turned out as the frequently occurring non-specific diseases [Table 1].

**Table 1:** Proportional incidence of diseases in dogs according to age.

Type of diseases	Diseases	Number of cases (Proportional incidence, %)				Chi-square value	P-value
		Puppy	Adolescent	Adult	Total		
Viral	Parvovirosis	51 (3.3)	23 (1.5)	62 (4.0)	136 (8.7)	7.54	0.02
	Distemper	20 (1.3)	14 (0.9)	11 (0.7)	45 (2.9)	12.19	0.00
Bacterial	Salmonellosis	21 (1.3)	16 (1.1)	25 (1.6)	62 (4)	2.37	0.31
	Colibacillosis	35 (2.2)	13 (0.8)	20 (1.3)	68 (4.4)	20.6	0.00
	Pneumonia	28 (1.8)	17 (1.1)	41 (2.6)	86 (5.5)	1.09	0.58
Parasitic	Helminthiasis	73 (4.7)	22 (1.4)	27 (1.7)	122 (7.8)	70.31	0.00
	Mange	23 (1.5)	31 (2.0)	94 (6.0)	148 (9.5)	14.98	0.00
	Myiasis	26 (1.7)	8 (0.5)	7 (0.4)	41 (2.6)	28.42	0.00
	Ectoparasitism	19 (1.2)	40 (2.6)	71 (4.6)	130 (8.3)	14.03	0.00
Sub-total		296 (19.0)	184 (11.81)	358 (23.0)	838 (53.8)		
Non-infectious	Lacerated wound	22 (1.4)	32 (2.1)	78 (5.0)	132 (8.5)	9.15	0.01
	Fracture	18 (1.1)	27 (1.7)	44 (2.9)	89 (5.7)	4.65	0.09
	Dystocia	0 (0.0)	0 (0.0)	22 (1.4)	22 (1.4)	22.46	0.00
	Abortion	0 (0.0)	0 (0.0)	16 (1.0)	16 (1.0)	16.47	0.00
	Partial paralysis	2 (0.1)	6 (0.4)	20 (1.3)	28 (1.8)	7.06	0.03
	Vit. deficiency	27 (1.7)	16 (1.1)	35 (2.4)	78 (5.0)	1.89	0.39
Sub-total		69 (4.4)	81 (5.2)	215 (13.8)	365 (23.4)		
Non-specific	Gastroenteritis	42 (2.7)	18 (1.2)	26 (1.7)	86 (5.5)	21.58	0.00
	Dermatitis	7 (0.4)	19 (1.2)	43 (2.8)	69 (4.4)	11.23	0.00
	Arthritis	3 (0.2)	6 (0.4)	28 (1.8)	37 (2.4)	11.01	0.00
	Orchitis	5 (0.3)	12 (0.8)	38 (2.4)	55 (3.5)	11.43	0.03
	Otorrhoea	2 (0.1)	5 (0.4)	11 (0.7)	18 (1.2)	1.29	0.53
	Alopecia	3 (0.2)	16 (1.0)	45 (2.9)	64 (4.1)	18.65	0.00
	Poisoning	7 (0.4)	6 (0.4)	12 (0.8)	25 (1.6)	0.05	0.98
Sub-total		69 (4.4)	82 (5.2)	203 (13.0)	354 (22.7)		
Grand total		434 (27.8)	347 (22.3)	776 (49.8)	1557 (100)		

**Figure 1:**
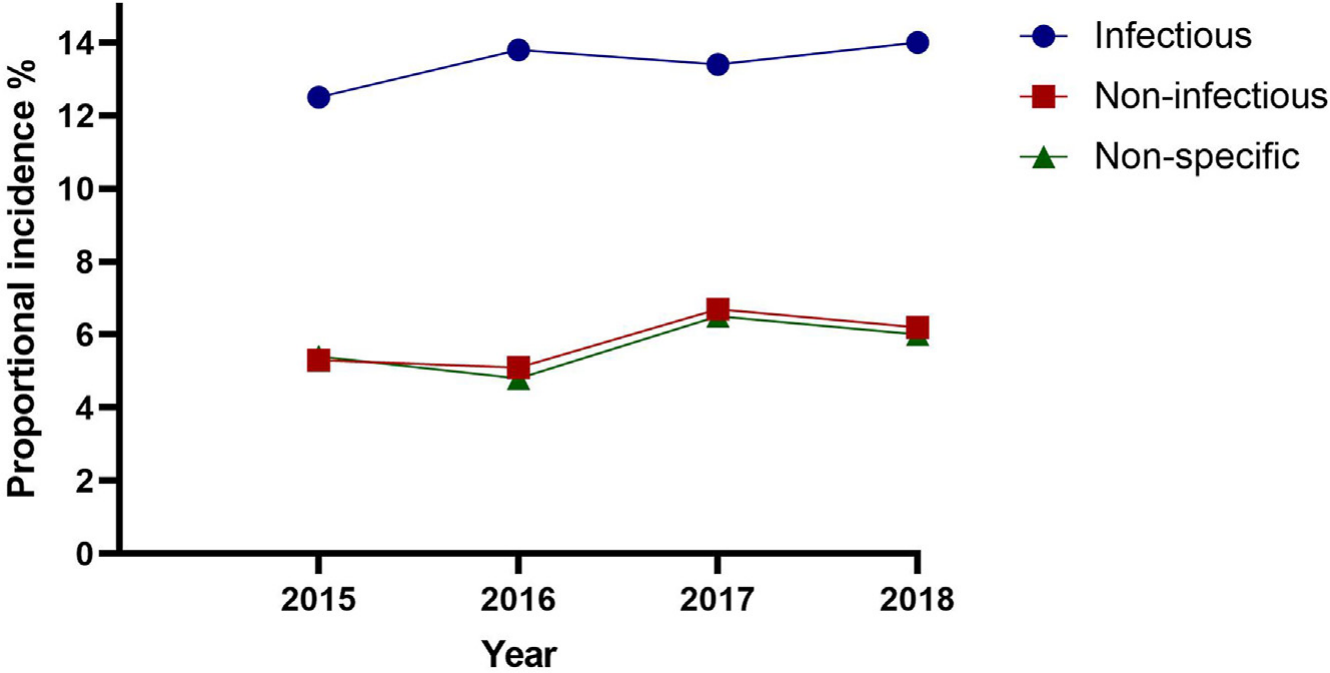
Year wise trends of the proportional incidence of infectious, non-infectious, and non-specific diseases.

Regarding the year wise distribution, the highest percentage of diseases was observed in the year 2017 (26.8%, n = 417) followed by 2018 (26.3%, n = 409), 2016 (23.8%, n = 371) and the lowest in 2015 (23.1%, n = 360) [Table 2]. Type wise disease trend according to the year indicated that infectious diseases had higher proportional incidence than non-infectious and non-specific diseases in all the years from 2015-2018. The year wise distribution of infectious diseases showed an upward trend, around 12.0% (2015) to around 14.0% (2018) while the non-infectious and non-specific diseases had a fluctuation in year wise proportional incidence, with the peak in 2017 for both types (non-infectious 6.7%, non-specific 6.5%) [Figure 1]. The proportional incidence of some specific diseases differed significantly (p < 0.05) among the years. The magnitude of parvovirosis (3.1%) and colibacillosis (1.6%) were higher in 2016 than other years; while gastroenteritis (2.2%) was more in 2017 compared to other years, and the proportional incidence varied significantly with year (p < 0.05) [Table 2].

**Table 2:** Proportional incidence of diseases in dogs according to year, 2015–2018.

Type of diseases	Diseases	Number of cases (Proportional incidence, %)					Chi-square value	P-value
		2015	2016	2017	2018	Total		
Viral	Parvovirosis	32 (2.1)	49 (3.1)	25 (1.6)	30 (1.9)	136 (8.7)	14.25	0.00
	Distemper	9 (0.6)	12 (0.8)	14 (0.9)	10 (0.6)	45 (2.9)	0.97	0.81
Bacterial	Salmonellosis	16 (1.0)	19 (1.2)	13 (0.9)	14 (0.9)	62 (4.0)	2.61	0.46
	Colibacillosis	13 (0.8)	25 (1.6)	16 (1.0)	14 (0.9)	68 (4.4)	6.64	0.00
	Pneumonia	22 (1.4)	20 (1.3)	28 (1.8)	16 (1.0)	86 (5.5)	3.42	0.33
Parasitic	Helminthiasis	23 (1.5)	30 (1.9)	37 (2.3)	32 (2.1)	122 (7.8)	1.09	0.64
	Mange	33 (2.1)	26 (1.7)	41 (2.6)	48 (3.1)	148 (9.5)	5.16	0.16
	Myiasis	12 (0.8)	10 (0.6)	8 (0.5)	11 (0.7)	41 (2.6)	1.53	0.68
	Ectoparasitism	35 (2.2)	24 (1.5)	28 (1.8)	43 (3.8)	130 (8.3)	6.56	0.09
Sub-total		195 (12.5)	215 (13.8)	210 (13.4)	218 (14.0)	838 (53.8)		
Non-infectious	Lacerated wound	30 (1.9)	27 (1.8)	40 (2.6)	35 (2.2)	132 (8.5)	1.37	0.71
	Fracture	17 (1.1)	20 (1.3)	28 (1.8)	24 (1.5)	89 (5.7)	1.52	0.68
	Dystocia	7 (0.4)	4 (0.3)	5 (0.3)	6 (0.4)	22 (1.4)	1.17	0.76
	Abortion	2 (0.1)	4 (0.3)	4 (0.3)	6 (0.4)	16 (1.0)	1.59	0.66
	Partial paralysis	4 (0.3)	8 (0.5)	10 (0.6)	6 (0.4)	28 (1.8)	2.37	0.51
	Vit. deficiency	23 (1.5)	17 (1.1)	18 (1.1)	20 (1.3)	78 (5.0)	2.02	0.57
Sub-total		83 (5.3)	80 (5.1)	105 (6.7)	97 (6.2)	365 (23.4)		
Non-specific	Gastroenteritis	15 (1.0)	13 (0.8)	35 (2.2)	23 (1.5)	86 (5.5)	10.76	0.01
	Dermatitis	15 (1.0)	23 (1.5)	13 (0.8)	18 (1.1)	69 (4.4)	4.49	0.21
	Arthritis	13 (0.8)	6 (0.4)	8 (0.5)	10 (0.6)	37 (2.4)	3.67	0.29
	Orchitis	17 (1.1)	13 (0.8)	15 (1.0)	10 (0.6)	55 (3.5)	2.92	0.40
	Otorrhoea	3 (0.1)	5 (0.4)	6 (0.4)	4 (0.3)	18 (1.2)	0.85	0.84
	Alopecia	14 (0.9)	10 (0.6)	21 (1.3)	19 (1.3)	64 (4.1)	3.13	0.37
	Poisoning	5 (0.3)	6 (0.4)	4 (0.3)	10 (0.6)	25 (1.6)	3.03	0.39
Sub-total		82 (5.4)	76 (4.8)	102 (6.5)	94 (6)	354 (22.7)		
Grand total		360 (23.1)	371 (23.8)	417 (26.8)	409 (26.3)	1557 (100)

**Figure 2:**
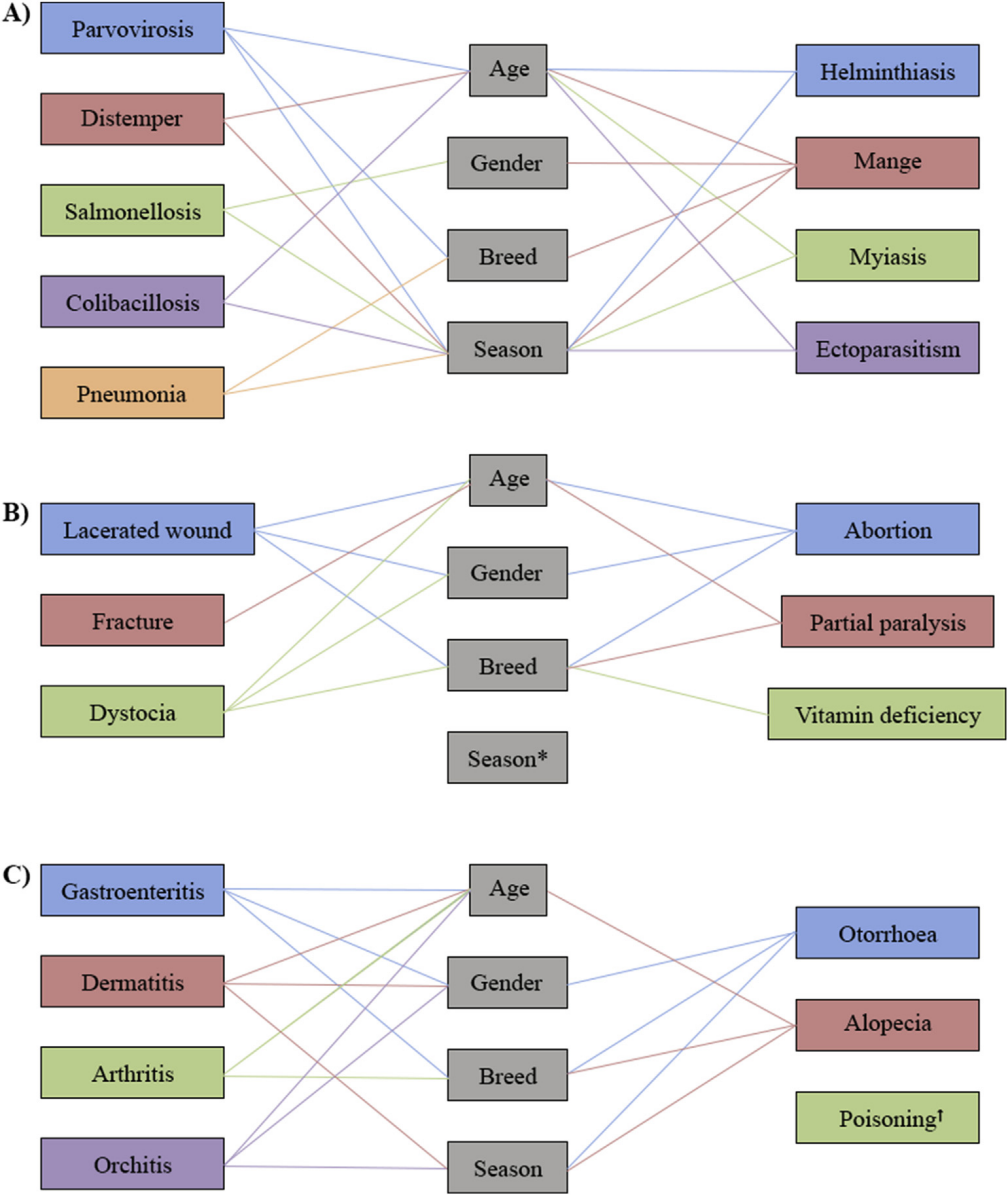
Illustration of variations in magnitudes of dog diseases among different levels of determinants (age, gender, breed, and season); A) Infectious diseases B) Non-infectious diseases C) Non-specific diseases ^‡^. ^‡^ A straight line connection between the disease and determinant/s denoted a significant variation in magnitudes of that disease among different levels of that/those determinants. ^∗^ No non-infectious diseases were found to have significant variations in magnitudes in terms of season. ^†^ Poisoning has no significant variations in magnitudes in term of any studied determinants.

The highest occurrence of diseases was observed in the adult dog (49.8%), then in the puppy (27.8%), and the lowest was in the adolescent (22.3%). In the adult, the magnitudes of parvovirosis (4.0%), mange (6.0%), ectoparasitism (4.6%), lacerated wound (5%), partial paralysis (1.3%), dermatitis (2.8%), arthritis (1.8%), orchitis (2.4%), and alopecia (2.9%) were higher than the puppy and adolescent, and they differed significantly (p < 0.05) among the age groups. Besides, distemper (1.3%), colibacillosis (2.6%), helminthiasis (4.7%), myiasis (1.7%), and gastroenteritis (2.7%) were more prevalent diseases in the puppy than other age groups, and the diseases showed a significant (p < 0.05) variation in proportional incidence among the age groups. However, few specific diseases were found to have a significant discrepancy in proportional incidence in the adolescent. Therefore, age was popped up as an important factor where the puppy and adult were the risk group in terms of disease occurrence [Table 1 and Figure 2].

According to the gender wise overall distribution of diseases in dogs, the burden in male (52.45%) was comparatively higher than in female (47.55%). In case of disease-specific proportional incidence by gender, salmonellosis (2.3%), gastroenteritis (3.2%), dermatitis (3.1%), and otorrhoea (0.8%) were significantly (p < 0.05) greater in female than male [Table 3 and Figure 2].

**Table 3:** Proportional incidence of diseases in dogs according to gender.

Type of diseases	Diseases	Number of cases (Proportional incidence, %)			Chi-square value	P-value
		Male	Female	Total		
Viral	Parvovirosis	77 (4.9)	59 (3.8)	136 (8.7)	0.30	0.32
	Distemper	23 (1.5)	22 (1.4)	45 (2.9)	0.03	0.86
Bacterial	Salmonellosis	25 (1.6)	37 (2.4)	62 (4.0)	3.78	0.05
	Colibacillosis	40 (2.6)	28 (1.8)	68 (4.4)	1.17	0.28
	Pneumonia	51 (3.1)	35 (2.4)	86 (5.5)	1.73	0.19
Parasitic	Helminthiasis	67 (4.3)	55 (3.5)	122 (7.8)	0.33	0.56
	Mange	81 (5.2)	67 (4.3)	148 (9.5)	0.35	0.05
	Myiasis	17 (1.1)	24 (1.5)	41 (2.6)	2.02	0.16
	Ectoparasitism	74 (4.8)	56 (3.5)	130 (8.3)	1.16	0.28
Sub-total		455 (29.2)	383 (24.6)	838 (53.8)		
Non-infectious	Lacerated wound	79 (5.1)	53 (3.4)	132 (8.5)	3.20	0.07
	Fracture	52 (3.3)	37 (2.4)	89 (5.7)	1.37	0.24
	Dystocia	0 (0.0)	22 (1.4)	22 (1.4)	24.57	0.00
	Abortion	0 (0.0)	16 (1.0)	16 (1.0)	17.80	0.00
	Partial paralysis	16 (1.1)	12 (0.7)	28 (1.8)	0.26	0.61
	Vit. deficiency	35 (2.4)	43 (2.6)	78 (5.0)	1.87	0.11
Sub-total		182 (11.7)	183 (11.8)	365 (23.4)		
Non-specific	Gastroenteritis	36 (2.3)	50 (3.2)	86 (5.5)	4.06	0.04
	Dermatitis	21 (1.3)	48 (3.1)	69 (4.4)	13.89	0.00
	Arthritis	12 (0.8)	25 (1.6)	37 (2.4)	6.06	0.14
	Orchitis	55 (3.5)	0 (0.0)	55 (3.5)	51.77	0.00
	Otorrhoea	5 (0.3)	13 (0.8)	18 (1.2)	4.43	0.04
	Alopecia	36 (2.3)	28 (1.8)	64 (4.1)	0.39	0.53
	Poisoning	14 (0.9)	11 (0.7)	25 (1.6)	0.13	0.72
Sub-total		179 (11.5)	175 (11.2)	354 (22.7)		
Grand total		816 (52.45)	741 (47.55)	1557 (100)		

Among the six types of dog breeds deemed under the current study, the highest overall proportional incidence was observed in the local breed (26.9%) followed by the German Shepherd (20%), the Labrador Retriever (17.5%), the Golden Retriever (13.6%), the Doberman (11.5%), and the Pomeranian breed (10.5%). In case of disease-specific proportional incidence by breed, the burden of parvovirosis (2.6%), mange (3.3%), lacerated wound (3.3%), and abortion (0.8%) was higher in the local breed than other breeds; accordingly partial paralysis (0.8%) and vitamin deficiency (1.2%) in the German Shepherd; otorrhoea (0.5%) in the Golden Retriever, pneumonia (1.7%) in the Doberman; dystocia (0.4%) and gastroenteritis (1.7%) in the Pomeranian breed; and a significant (p < 0.05) variation in the proportional incidence of those specific diseases was obtained among the dog breeds, indicating breed as a key determinant that could modify the occurrence of some specific diseases in dogs [Table 4 and Figure 2].

**Table 4:** Proportional incidence of diseases in dog according to breed.

Type of diseases	Diseases	Number of cases (Proportional incidence, %)							Chi-square value	P-value
		GS	LR	GR	D	P	L	Total		
Viral	Parvovirosis	30 (1.9)	21 (1.3)	12 (0.7)	24 (1.6)	8 (0.6)	41 (2.6)	136 (8.7)	11.64	0.04
	Distemper	8 (0.6)	9 (0.6)	6 (0.4)	5 (0.3)	2 (0.1)	15 (0.9)	45 (2.9)	2.61	0.76
Bacterial	Salmonellosis	13 (0.8)	8 (0.6)	4 (0.3)	10 (0.6)	8 (0.6)	19 (1.1)	62 (4.0)	5.12	0.39
	Colibacillosis	15 (0.9)	11 (0.7)	13 (0.8)	9 (0.6)	7 (0.6)	13 (0.8)	68 (4.4)	3.87	0.56
	Pneumonia	12 (0.7)	11 (0.7)	7 (0.5)	26 (1.7)	14 (0.9)	16 (1.0)	86 (5.5)	37.89	0.00
Parasitic	Helminthiasis	19 (1.1)	23 (1.5)	20 (1.3)	15 (0.9)	17 (1.1)	28 (1.9)	122 (7.8)	4.58	0.47
	Mange	35 (2.3)	27 (1.7)	15 (0.9)	12 (0.7)	8 (0.6)	51 (3.3)	148 (9.5)	15.57	0.00
	Myiasis	8 (0.6)	5 (0.3)	4 (0.3)	5 (0.3)	2 (0.1)	17 (1.0)	41 (2.6)	5.73	0.33
	Ectoparasitism	21 (1.3)	25 (1.6)	17 (1.0)	19 (1.1)	9 (0.6)	39 (2.7)	130 (8.3)	4.76	0.45
Sub-total		161 (10.3)	140 (9.0)	98 (6.2)	125 (8.0)	75 (4.9)	239 (15.0)	838 (53.8)		
Non-infectious	Lacerated wound	26 (1.8)	21 (1.3)	19 (1.1)	8 (0.6)	6 (0.4)	52 (3.3)	132 (8.5)	17.17	0.00
	Fracture	18 (1.1)	15 (0.9)	12 (0.7)	8 (0.6)	10 (0.6)	26 (1.8)	89 (5.7)	0.78	0.98
	Dystocia	4 (0.3)	3 (0.2)	4 (0.3)	2 (0.1)	7 (0.4)	2 (0.1)	22 (1.4)	13.03	0.02
	Abortion	4 (0.3)	0 (0.0)	0 (0.0)	0 (0.0)	0 (0.0)	12 (0.8)	16 (1.0)	22.07	0.00
	Partial paralysis	12 (0.8)	4 (0.3)	3 (0.2)	0 (0.0)	0 (0.0)	9 (0.6)	28 (1.8)	14.30	0.01
	Vit. deficiency	19 (1.2)	12 (0.7)	17 (1.0)	7 (0.6)	13 (0.8)	10 (0.6)	78 (5.0)	14.53	0.01
Sub-total		83 (5.3)	55 (3.5)	55 (3.5)	25 (1.7)	36 (2.3)	111 (7.1)	365 (23.4)		
Non-specific	Gastroenteritis	12 (0.7)	15 (0.9)	10 (0.6)	7 (0.6)	27 (1.7)	15 (1.0)	86 (5.5)	43.95	0.00
	Dermatitis	12 (0.7)	17 (1.0)	13 (0.8)	8 (0.6)	4 (0.3)	15 (1.0)	69 (4.4)	6.05	0.30
	Arthritis	7 (0.6)	11 (0.7)	9 (0.6)	3 (0.2)	2 (0.1)	5 (0.3)	37 (2.4)	10.31	0.07
	Orchitis	10 (0.6)	8 (0.6)	5 (0.3)	7 (0.6)	8 (0.6)	17 (0.8)	55 (3.5)	2.55	0.77
	Otorrhoea	2 (0.1)	7 (0.5)	8 (0.6)	0 (0.0)	0 (0.0)	1 (0.1)	18 (1.2)	25.30	0.00
	Alopecia	19 (1.2)	16 (1.0)	9 (0.6)	3 (0.2)	5 (0.3)	12 (0.8)	64 (4.1)	10.07	0.07
	Poisoning	6 (0.4)	3 (0.2)	5 (0.3)	1 (0.1)	6 (0.4)	4 (0.2)	25 (1.6)	8.20	0.15
Sub-total		68 (4.4)	77 (4.9)	59 (3.8)	29 (1.9)	52 (3.3)	69 (4.4)	354 (22.7)		
Grand total		312 (20.0)	272 (17.5)	212 (13.6)	179 (11.5)	163 (10.5)	419 (26.9)	1557 (100)		

GS- German Sheperd; LR- Labrador Retriever; GR- Golden Retriever; D- Doberman; P- Pomeranian; L- Local breed.

According to the season, overall, most of the diseases were commonly observed in the summer season (35.0%), next of the rainy season (32.5%), and the winter season (32.5%). However, the present study disclosed that magnitudes of several specific diseases varied significantly among the summer, winter, and rainy season. In the winter season, the proportional incidence of parvovirosis (4.7%), pneumonia (2.6%), dermatitis (2.4%), and alopecia (2.2%) were significantly higher than the summer and rainy season (p < 0.05). Besides, the proportional incidence of salmonellosis (2.4%), colibacillosis (2.7%), mange (4.4%), and myiasis (1.4%) were significantly higher in the summer season than the rainy and winter season (p < 0.05). Likely, the proportional incidence of helminthiasis (3.6%), ectoparasitism (5%), and orchitis (2%) were significantly higher in the rainy season than in other seasons (p < 0.05) [Table 5 and Figure 2]. These findings showed that the season was an important determinant for the occurrence of several dog diseases.

**Table 5:** Proportional incidence of diseases in dog according to season.

Type of diseases	Diseases	Number of cases (Proportional incidence, %)				Chi-square value	P-value
		Winter	Summer	Rainy	Total		
Viral	Parvovirosis	73 (4.7)	26 (1.7)	37 (2.4)	136 (8.7)	32.4	0.00
	Distemper	22 (1.4)	11 (0.7)	12 (0.8)	45 (2.9)	5.74	0.06
Bacterial	Salmonellosis	11 (0.7)	37 (2.4)	14 (0.9)	62 (4.0)	17.52	0.00
	Colibacillosis	10 (0.6)	42 (2.7)	16 (1.0)	68 (4.4)	22.25	0.00
	Pneumonia	41 (2.6)	17 (1.1)	28 (1.8)	86 (5.5)	12.42	0.00
Parasitic	Helminthiasis	27 (1.7)	39 (2.5)	56 (3.6)	122 (7.8)	12.17	0.00
	Mange	47 (3.0)	69 (4.4)	32 (2.1)	148 (9.5)	12.24	0.00
	Myiasis	7 (0.4)	22 (1.4)	12 (0.8)	41 (2.6)	7.42	0.02
	Ectoparasitism	18 (1.2)	34 (2.2)	78 (5.0)	130 (8.3)	51.66	0.00
Sub-total		256 (16.4)	297 (19)	285 (18.3)	838 (53.8)		
Non-infectious	Lacerated wound	45 (2.9)	54 (3.5)	33 (3.1)	132 (8.5)	3.99	0.14
	Fracture	25 (1.6)	37 (2.4)	27 (1.7)	89 (5.7)	1.87	0.39
	Dystocia	8 (0.6)	6 (0.2)	8 (0.6)	22 (1.4)	0.5	0.75
	Abortion	7 (0.5)	3 (0.2)	6 (0.3)	16 (1.0)	1.97	0.37
	Partial paralysis	10 (0.6)	11 (0.7)	7 (0.5)	28 (1.8)	0.72	0.69
	Vit. deficiency	21 (1.3)	26 (1.6)	31 (2.1)	78 (5.0)	2.22	0.33
Sub-total		116 (7.4)	137 (8.7)	112 (7.1)	365 (23.4)		
Non-specific	Gastroenteritis	28 (1.8)	33 (2.1)	25 (1.6)	86 (5.5)	0.61	0.74
	Dermatitis	37 (2.4)	18 (1.2)	14 (0.9)	69 (4.4)	14.76	0.00
	Arthritis	12 (0.7)	18 (1.2)	7 (0.5)	37 (2.4)	4.15	0.13
	Orchitis	11 (0.7)	13 (0.8)	31 (2.0)	55 (3.5)	14.93	0.00
	Otorrhoea	2 (0.1)	6 (0.5)	10 (0.6)	18 (1.2)	4.69	0.09
	Alopecia	35 (2.2)	17 (1.1)	12 (0.8)	64 (4.1)	12.24	0.00
	Poisoning	10 (0.6)	6 (0.5)	9 (0.5)	25 (1.6)	1.41	0.49
Sub-total		135 (8.6)	111 (7.1)	108 (6.9)	354 (22.7)		
Grand total		507 (32.5)	545 (35.0)	505 (32.5)	1557 (100)		

## 4 Discussion

We calculated the proportional incidence from hospital-based registered data. Therefore, it might happen that few dog cases which had not been attended to the hospital by owners were not included in the study. The study highlighted the magnitudes of dog diseases in terms of several key determinants. The study revealed that magnitudes of infectious diseases were higher than non-infectious and non-specific cases. The findings corroborated the previous study of Sharma et al. (2008) who reported that dogs were mostly affected by infectious diseases (70.90% of total cases), rather than by non-infectious and non-specific diseases in New Delhi, India. A previous study reported around 18.5 thousand free roaming dog population in the Dhaka city and two-thirds of them were unvaccinated against different infectious diseases and they were deprived of sanitation and hygienic management, which triggered the transmission of infectious diseases among dogs (Tenzin et al., 2015). Besides, the exposure of the pet dogs with this vast amount of free roaming street dog population, who are believed to be potential spreaders of infectious diseases, might be a reason of higher incidence of infectious disease than non-infectious and non-specific diseases in the current study. Among infectious cases, parasitic diseases had higher proportional incidence than viral and bacterial diseases. This finding coincided with the previous study of William et al. (2002) who reported around 46.0% of the total diseases were parasitic diseases in dogs in Nigeria. Besides, higher proportional incidence of mange than other parasitic diseases observed in this study agreed with the findings of Sharma et al. (2008) who reported the higher occurrence of mange in dogs in comparison to other parasitic diseases and demonstrated that contaminated beddings and furniture of pets and poor hygiene as risk factors for the occurrence of the disease, and these factors could also be the reason of a high occurrence level of mange in the Dhaka city. Accordingly, the proportional incidence of parvovirosis was higher than other viral diseases, which showed a semblance with the findings of Behera et al. (2015) where they reported 40.5% of viral diseases was attributed due to parvovirosis in Odisha, India. A previous study showed that most of the stray dogs in the Dhaka city were unvaccinated against parvovirosis (Tenzin et al., 2015) and lack of vaccination was identified as a key determinant of the occurrence of the disease in dogs by Roy et al. (2018). These circumstances might attribute to a high burden of parvovirosis in the Dhaka city. The overall proportional incidence of pneumonia was higher than other bacterial diseases, which was supported by previous study Hossain et al. (2017) targeting dogs in the Sylhet district of Bangladesh. Moreover, the proportional incidence of lacerated wound and gastroenteritis were higher than other non-infectious cases and non-specific cases, respectively. These findings of the present study matched with the findings of Shima et al. (2015a) where 7.0% of non-specific diseases were gastroenteritis.

Regarding the age wise distribution of the diseases in dogs, the highest percentage was observed in the adult followed by the puppy and adolescent, which was consistent with the study of Hossain et al. (2017) who reported 47.5% of the total diseases occurred in the adult dog. The magnitudes of parvovirosis, wounds, abortion, dystocia, partial paralysis, mange, and ectoparasitic infection had a significant (p < 0.05) age wise variation and the adults were mostly prone to get infected by those diseases. Higher occurrence of wound in the adult dogs than other age groups might be due to injury by road accident, malicious action, and defensive or territorial manners of them (Shima et al., 2014). Abortion and dystocia were significant in adult age due to attaining their genderual maturity. Breed variation and infections with various pathogenic microorganisms particularly protozoa may cause abortion in dog (Endrias et al., 2010). Distemper, colibacillosis, helminthiasis, and myiasis showed a significant (p < 0.05) difference in proportional incidence among the age groups, and mostly occurred in the puppy compared to other groups. The existing findings were consistent with the previous study of Tarafder and Samad (2010) in Bangladesh who reported 1.47% prevalence of canine distemper in the puppy up to 6 months of age in comparison to 7–36 months (0.14%) and above 36 months (0.0%) age groups of dogs. Colibacillosis occurred at high frequency in the puppy, which was persistent with the earlier study targeting dogs in Canada (Broes et al., 1988). In the same way, puppies were mostly prone to helminthiasis than adults, which was bolstered by Endias et al. (2010) and Muradian et al. (2005). They reported that enteric helminthiasis occurred more in puppies than adults in Ethiopia and Brazil, respectively.

Regarding the gender wise distribution of diseases, the proportional incidence in male dogs was higher than that in female dogs, which conformed the findings of Yadav et al. (2017) in the Chittagong city of Bangladesh and Behera et al. (2015) in Odisha, India. According to this study, female was more prone to gastroenteritis than male, showing a contradiction with the findings of Ide et al. (2002) who found male suffers more from gastroenteritis than female due to higher oxidative stress. However, in the current study female might have a high exposure to different causative agents of gastrointestinal diseases, and immune insufficiency during the pregnancy period may increase the propensity to a high frequency of gastroenteritis in them. In the existing study, dermatitis tended to be higher in female than male, which might be due to the contact with different skin irritants and external parasitic infestation. However, several prior researches didn't find any association of gender with dermatitis (Bizikova et al., 2015; Bruet et al., 2012).

According to the breed wise overall distribution of diseases in dogs, the local breed was higher in disease occurrence than other breeds, which corroborated the previous studies conducted in the Sylhet district of Bangladesh where they found 31.57% of total diseased dogs were local breed (Hossain et al., 2017). According to Khare et al. (2019), the magnitude of canine parvovirus in dog was higher in local breed than other exotic breeds in Jabalpur, India, which also supported the findings of the current study. The previous research demonstrated that lack of vaccination, lack of nutrition, and poor health status act as the major risk factors for parvovirosis in dogs (Naveenkumar et al., 2019). Usually, the local dog breed is reared by comparatively impoverished owners in the Dhaka city. So, they could not provide sufficient veterinary care and nutritious diets to dogs, which might be the reason for higher parvovirosis incidence in local breed than exotic breeds. Mange, lacerated wound, and abortion had a significant (p < 0.05) breed wise variation in terms of proportional incidence, and these diseases occurred more in local breed than other breeds. Yadav et al. (2017) also reported that the occurrence of mange was greater in local breed than other exotic breeds, which supported the findings of the present study. The existing findings might be due to exotic breeds were mostly reared with proper care, but the local breeds did not get such intensive care. Besides, local breed had higher contacts with street dogs than other breeds, which might facilitate the transmission of ticks and mites to them, causing the occurrence of mange at a high frequency. The predominant causes of wounds were road accident, fighting, and human cruelty and the likelihood of happenings of those incidents were reported to be higher in the local breed than others, as they were kept free (Shima et al., 2014). Besides, this free ranging rearing of local dog breed increased the chance of exposures to various pathogens causing abortion in them compared to other breeds, which might be a reason for such findings (Endrias et al., 2010). The long ear of Golden Retriever might be a special reason for susceptibility to otorrhoea; whilst the likelihood of occurrence of dystocia in the Pomeranian breed was high for their tiny anatomical conformation (Ludwig, 2008).

According to the overall seasonal proportional incidence, the highest percentage of dog diseases was observed in the summer than other seasons, which contrasted the findings of Tarafder and Samad (2010) who found the highest prevalence of dog diseases in the winter (29.84%). Parvovirosis, pneumonia, dermatitis, and alopecia showed a significant (p < 0.05) difference in seasonal proportional incidence and those diseases tended to occur more in the winter compared to other seasons. These findings were consistent with the previous study by Tarafder and Samad (2010) who stated that proportional incidence of parvovirus and pneumonia were higher in the winter than other seasons. Besides, cold weather might worsen the immune system and stimulate the growth and multiplication of various pathogens responsible for causing pneumonia (Heikki, 2007). Moreover, higher amount of dust in the winter than other seasons might act as an important environmental allergen associated with dermatitis (De Weck, 1995; Lian and Halliwell, 1998). Salmonellosis, colibacillosis, mange, and myiasis also had a significant (p < 0.05) seasonal variation in proportional incidence, however, they were more frequent in the summer than other seasons. The reason of a high occurrence level of salmonellosis in the summer season could be due to the favorable environmental effect for promoting the growth and multiplication of salmonella organisms (Reimschuessel et al., 2017). Besides, the hot and humid climate during the summer season could facilitate multiplication, spread, and survival of most of the vector flies, which might attribute to the high proportional incidence of mange in this season (Dewan and Yamaguchi, 2009), while untreated wound, matted hair, and owner ignorance may predispose to myiasis infestation (Anderson and Huitson, 2004). A significant seasonal variation in proportional incidence was also observed in case of ectoparasitism, helminthiasis, and orchitis, and they were found to be occurred at greater amount in the rainy season than other seasons. Probably, the wet environmental conditions of the rainy season favored the breeding and hatchability of the parasites responsible for high degrees of ectoparasitism and helminthiasis (Shima et al., 2015a). However, the findings of this study disagreed with Lyronis et al. (2009) who reported that orchitis had no seasonal significance in terms of prevalence. Nevertheless, Sen Majumder and Bhadra (2015) revealed a strong correlation between the season and mating of free range dogs in India. They found that during the rainy season, increased environmental humidity and low temperature triggered pheromone signals in dogs that intensified their sexual responses. Additionally, unprotected sexual intercourses in dogs could predispose them to sexually transmitted diseases, reported by Tarafder and Samad (2010) in Bangladesh. So, these evidences might be reflected in this study and the rainy season had been appeared to have a significant association with a high frequency level of incidence of orchitis in dogs.

In the current study, passive data were used from the hospital registered book. So, relatively more accurate scenario could be found if the study would be performed with data by an active survey. Moreover, the diagnostic tests used in the study had high sensitivities and specificities. In spite of that, there might have some information biases, but rationally they were supposed to be very negligible. Despite these limitations, we believed that the findings of this study were reliable and could be used for further references.

## 5 Conclusions

The current study provides basic information regarding the proportional incidence of dog diseases and their distribution in terms of age, gender, breed, and season. Mange showed a significant variation in magnitudes in case of all studied determinants. Besides, significant discrepancies in magnitudes of lacerated wound, dystocia, abortion, and gastroenteritis were found among various groups of age, gender, and breed. In the same way, dermatitis and orchitis had significant differences in proportional incidence in case of age, gender, and season; whilst the burden of parvovirosis and alopecia differed significantly amongst categories of age, breed, and season. Accordingly, the magnitude of otorrhoea showed a significant variation among different groups of gender, breed, and season. Hence, the findings of this study could help owners employ disease-specific special cares and control measures to the vulnerable levels of the studied determinants at the right time, like dedicated vaccination for the puppies and adults prior to the winter season in case of parvovirosis. On top of this, the current findings could also be used as baseline information for further researches and strategic planning to prevent and control those diseases.

## Declarations

### Author contribution statement

Mohammod Misbah Uddin: Conceived and designed the experiments; Analyzed and interpreted the data; Wrote the paper.

Himel Talukder: Analyzed and interpreted the data; Wrote the paper.

Obaidul Islam, Md. Asaduzzaman, Moumita Das: Analyzed and interpreted the data.

Md. Irtija Ahsan: Conceived and designed the experiments; Wrote the paper.

Saiful Islam: Conceived and designed the experiments.

### Funding statement

This research did not receive any specific grant from funding agencies in the public, commercial, or not-for-profit sectors.

### Data availability statement

Data included in article/supplementary material/referenced in article.

### Declaration of interests statement

The authors declare no conflict of interest.

### Additional information

No additional information is available for this paper.
